# Cardiometabolic indices predict hypogonadism in male patients with type 2 diabetes

**DOI:** 10.1007/s40618-022-01941-0

**Published:** 2022-10-25

**Authors:** N. Caretta, P. Facondo, S. Mereu, A. Delbarba, M. C. Crepaldi, M. Vedovato, A. Avogaro, A. Ferlin

**Affiliations:** 1 Unit of Andrology and Reproductive Medicine, University Hospital of Padova, Padua, Italy; 2 Department of Clinical and Experimental Sciences, University of Brescia, Brescia, Italy; 3 Department of Medicine, University of Padova, Padua, Italy; 4 Unit of Endocrinology and Metabolism, ASST Spedali Civili, Brescia, Italy; 5 Department of Medicine, Division of Metabolic Diseases, University of Padova, Padua, Italy; 6 Department of Medicine, Unit of Andrology and Reproductive Medicine, University of Padova, Padua, Italy

**Keywords:** Male hypogonadism, Metabolic syndrome, Testosterone, Type 2 diabetes

## Abstract

**Purpose:**

To evaluate in men with type 2 diabetes the association of cardiometabolic indices [Visceral Adiposity Index (VAI), Triglyceride Glucose Index (TyG), and lipid accumulation product (LAP)] with total testosterone (TT) levels, and their predictive cut-off values in identifying hypogonadism.

**Methods:**

265 consecutive men aged 40–70 years with type 2 diabetes performed an andrological evaluation; metabolic parameters and TT were determined. Receiver operating characteristic (ROC) curves were used to identify cut-off values of cardiometabolic indices in predicting low testosterone (TT < 12 nmol/l).

**Results:**

VAI, TyG, and LAP were negatively associated with TT levels. The prevalence of hypogonadism in men in the fourth quartiles of VAI, TyG, and LAP was ~ 70.0–75.0% compared to ~ 10.0–17.0% in men in the first quartiles (*p* < 0.001). The sensitivity and specificity of the three cardiometabolic indices in predicting TT < 12 nmol/l were significantly higher concerning BMI, waist circumference, lipid profile and HbA1c. Cut off values of VAI ≥ 3.985, TyG ≥ 4.925, and LAP ≥ 51.645 predict hypogonadism with good sensitivity and specificity.

**Conclusion:**

This is the first study evaluating the association of VAI, TyG, and LAP with hypogonadism in men with type 2 diabetes. Alterations in these indices should direct the patients to andrological evaluation.

**Supplementary Information:**

The online version contains supplementary material available at 10.1007/s40618-022-01941-0.

## Introduction

Male hypogonadism is defined as low circulating testosterone (T) level associated with signs and symptoms of testosterone deficiency, caused by organic or functional defects at multiple levels of the hypothalamic-pituitary–gonadal axis [1]. Functional hypogonadism, a potentially reversible impairment of the hypothalamic–pituitary–testis axis, is the most frequent form in adult men (> 40–50 years): this condition is associated mainly with comorbid conditions, such as chronic diseases (heart, liver, kidney failure), obesity, metabolic syndrome, and type 2 diabetes mellitus [2, 3].

In particular, it is well known that patients with type 2 diabetes are at high risk of hypogonadism. As early as 1990 [4], many studies reported T levels significantly lower and the prevalence of hypogonadism substantially higher with respect to men without diabetes, particularly in men with obesity, insulin resistance, and metabolic syndrome [5]. In most studies, patients with type 2 diabetes have significantly lower total T (TT), free T (FT), and sex-hormone binding globulin (SHBG), with low or inappropriately normal luteinizing hormone (LH) plasma levels, a condition known as hypogonadotropic hypogonadism [5, 6]. A recent meta-analysis including 56 studies and 6856 patients with type 2 diabetes and 23,572 controls confirmed significant decreases in TT (not associated with age or body mass index [BMI]), FT, and SHBG levels [7].

The bidirectional association of T concentration with adiposity and insulin resistance is also well known, and men with hypogonadism are at risk of developing metabolic syndrome and type 2 diabetes [5, 6]. In men with type 2 diabetes, testosterone treatment aimed to improve the anthropometric and glyco-metabolic parameters is debated [8]; otherwise, it is recommended in diabetic patients with reduced serum TT levels (< 12 nmol/l) associated with symptoms of sexual dysfunction (erectile dysfunction [ED], decreased libido and morning erection) [9], as testosterone therapy improves disease control and outcome [10, 11].

Nevertheless, the following aspects need to be considered: (1) diabetologists are poorly used to discuss sexual health with their patients and assess testis endocrine function [12]; (2) in men with type 2 diabetes, sexual dysfunctions (as ED) might also be related to poor metabolic control, arteriopathy and autonomic neuropathy other than to T deficiency [13]; (3) a diagnosis of hypogonadism based on symptoms may be challenging to make in men with type 2 diabetes. Indeed, symptoms of T deficiency (as decreased energy, motivation, physical strength, depressed mood, poor concentration, and memory, reduced muscle mass and strength, and increased body fat/central obesity) could overlap diabetes and hypogonadism, leading difficult to suspect the patient’s hypogonadism [12].

Therefore, an important issue is the identification of clinical or biochemical parameters that diabetologists may routinely use to predict hypogonadism in men with type 2 diabetes and to suggest an andrological evaluation. In this context, common cardiometabolic indices as Visceral Adiposity Index (VAI) [14], Triglyceride Glucose Index (TyG) [15] and Lipid Accumulation Product (LAP)] [16] can be a mirror of insulin sensitivity [17], glyco-metabolic control [18] and sexual health [19–21] and may predict hypogonadism in men without diabetes [22, 23]. However, to the best of our knowledge, no study has evaluated this aspect in men with type 2 diabetes.

So, in this study, we aimed to evaluate, in men with type 2 diabetes, the association of VAI, TyG and LAP with T plasma levels, and to assess their predictive cut-off values in identifying hypogonadism.

## Methods

We prospectively enrolled 265 consecutive men affected by type 2 diabetes, followed at the Division of Metabolic Diseases of the University Hospital of Padova (Italy) and evaluated at the Unit of Andrology and Reproductive Medicine of the same Hospital from 1 January 2016 to 31 December 2019. We enrolled men between 40 and 70 years without uncompensated glycemic symptoms (polyuria, polydipsia, weight loss) and overt diabetic acute or chronic complications. In detail, exclusion criteria were: no previous or current cardiovascular events (myocardial infarction, heart failure, stroke, obliterating arterial or thromboembolic disease), overt diabetic retinopathy, overt diabetic nephropathy (excluding microalbuminuria), clinical signs of diabetic neuropathy, hyperosmolar hyperglycemic syndrome, known forms of organic hypogonadism (such as Klinefelter syndrome, Kallmann syndrome, cryptorchidism, testicular trauma or torsion or cancer, orchitis, pituitary disorders, HIV [Human Immunodeficiency Virus], tumors, organ failure, chemo- and radio- therapy, hormonal treatment or drugs interfering with testosterone levels). Glycated hemoglobin (HbA1c) levels, diabetes duration and the type of anti-hyperglycemic treatment did not represent selection criteria.

All subjects performed a complete andrological assessment with history, physical (including waist circumference [wc], weight and height measurement to calculate BMI) and testicular examination (to assess volumes through Prader’s orchidometer), and IIEF-5 (International Index of erectile function-5), IPSS (International Prostatic Symptoms Score) and AMSS (Aging Male Symptom Score) questionnaires. IIEF-5 questionnaire is used to assess the erectile function and to identify the presence of ED for values ≤ 21 [24], IPSS questionnaire to evaluate the presence of low urinary tract symptoms [25], and AMSS questionnaire to assess the absence (AMSS ≤ 26) or presence of signs compatible with mild (AMSS 27–36), moderate (AMSS 37–49) or severe (AMSS ≥ 50) T deficiency [26].

Blood samples in fasting morning between 8:00 am and 10:00 am were collected for the following biochemical assays: glycemia, HbA1c, lipid profile (total, HDL [high-density lipoprotein], non-HDL cholesterol, triglycerides [TG]), creatinine, TT, LH, estradiol, prostate-specific antigen (PSA) and 25-OH vitamin D. To reduce variability in the determination of TT and estradiol assay, only the samples analyzed at the central laboratory of University Hospital of Padova (chemiluminescence microparticle immunoassay) were considered (*n* = 190).

VAI, TyG and LAP have been calculated using automatic online calculators with the following formula [14–16]: VAI = wc/[39.68 + (1.88 × BMI)] × TG/1.03 × 1.31/HDL; TyG = log [glycemia (mg/dL) × TG (mg/dL)/2]; LAP = (wc − 65) × TG. Plasma concentration of TT have been considered normal (≥ 12 nmol/l), low (8–12 nmol/l) and frankly low (< 8 nmol/l), according to guidelines on functional hypogonadism [2]. Levels of LH > 9.4 UI/l have been considered as elevated [27].

The study protocol follows the standard clinical approach and the principles outlined in the Declaration of Helsinki. Informed consent to collect the data anonymously for the scientific purpose was obtained from the study participants.

Statistical Package for the Social Sciences software IBM SPSS Statistics, Version 26.0, Armonk (NY) was used for statistical analysis. Since the variables were not normally distributed (by Kolmogorov–Smirnov test), continuous variables were expressed as median (and interquartile range), while categorical were as percentage. Correlation analyses between clinical and biochemical data were performed using Pearson’s correlation (for continuous variables) and point-biserial correlation (for categorial/dichotomous variables). Within the population analyzed (subdivided by values of TT and by quartiles of VAI, TyG, and LAP), comparisons between clinical and biochemical variables were performed by non-parametric tests, in particular Mann–Whitney test (for continuous variables) and Pearson’s Chi-Square or Kruskal–Wallis tests (for categorical variables). To determine factors associated with TT < 12 nmol/l, multivariate (preceded by univariate) correlation analyzes were performed, using binary linear logistic regression. At binary linear logistic regression, the analyses were carried out between variables, considering the indices (VAI, TyG, and LAP) and their constituent parameters. ROC (Receiver operating characteristic) curves were performed and analyzed to assess sensitivity, specificity and optimal cut-off of the different variables in predicting TT < 12 nmol/l. AUC (area under the curve) < 0.5 was considered as unable to discriminate patients with TT < 12 nmol/l. To establish the factors more predictive for TT < 12 nmol/l, ROC curves were compared by *z*-test. A *p* value < 0.05 was considered significant.

## Results

Table 1 reports the clinical and biochemical data of overall patients (*n* = 265, of which 190 with available T levels). Based on IIEF-5, 68.1% of patients had ED and according to AMSS 36.8%, 27.2% and 5.3% of men had symptoms compatible with mild, moderate and severe androgen deficiency, respectively.

**Table 1:** Clinical and biochemical data of patients and comparison between men with frankly low vs low vs normal TT (nmol/l)

	Overall	TT < 8 (*n* = 25)	8 ≤ TT < 12 (*n* = 52)	TT ≥ 12 (*n* = 113)	*p*
Age (year)	58.0 (51.0–64.0)	59.0 (54.0–65.0)	57.5 (48.7–62.7)	58.0 (51.0–65.0)	0.583
BMI (kg/m^2^)	28.4 (25.8–31.1)	31.4 (29.6–34.9)	28.8 (26.8–32.1)	27.9 (25.0–30.7)	**0.008**
Waist circumference (cm)	101.0 (94.0–109.2)	115.0 (105.5–118.5)	102.5 (96.2–111.0)	99.0 (92.7–107.2)	**< 0.001**
Glycemia (mg/dl)	142.0 (122.0–175.0)	159.0 (137.0–184.0)	154.0 (126.7–183.2)	137.0 (120–0-164.0)	**0.024**
HbA1c (%)	7.2 (6.6–8.1)	7.4 (6.9–8.5)	7.3 (6.6–8.6)	6.9 (6.5–7.8)	**0.0028**
Total cholesterol (mg/dl)	174.0 (154.0–197.0)	189.0 (163.0–2225.0)	175.0 (156.2–200.2)	173.0 (157.0–192.0)	0.189
HDL cholesterol (mg/dl)	44.0 (38.0–54.0)	41.0 (33.0–50.0)	40.5 (35.0–44.2)	46.0 (40.0–56.0)	**0.021**
Non-HDL cholesterol (mg/dl)	113.0 (87.8–131.0)	121.0 (96.4–138.0)	122.0 (97.2–153.0)	112.0 (89.8–129.0)	0.067
Triglycerides (mg/dl)	121.0 (87.0–187.0)	177.0 (143.0–245.0)	164.5 (114.7–216.2)	101.0 (82.0–132.0)	**< 0.001**
Creatinine (mg/dl)	0.8 (0.7–0.9)	0.9 (0.7–1.0)	0.9 (0.8–1.0)	0.8 (0.7–0.9)	0.380
LH (IU/l)	5.0 (3.8–6.9)	5.7 (4.2–9.1)	5.4 (4.0–8.0)	5.0 (3.8–6.2)	0.324
TT (nmol/l)	12.9 (10.2–16.6)	6.8 (5.6–7.8.0)	9.2 (8.7–11.4)	15.3 (13.1–18.7)	**< 0.001**
E2 (pmol/l)	82.0 (55.2–105.0)	54.0 (39.0–87.2)	84.0 (59.5–101.5)	86.5 (59.2–109.0)	0.132
PSA (ng/ml)	0.9 (0.5–1.6)	0.7 (0.3–1.0)	0.8 (0.5–1.6)	1.0 (0.6–1.9)	0.161
25 OH-Vitamin D (nmol/l)	33.5 (20.0–49.0)	21.5 (15.7–31.5)	33.0 (17.5–45.0)	38.0 (23.5–49.5)	**0.016**
Right testicular volume (ml)	14.0 (12.0–16.7)	14.0 (11.0–16.0)	16.0 (13.6–18.0)	14.0 (12.0–18.0)	0.682
Left testicular volume (ml)	14.0 (12.0–16.0)	13.5 (11.0–16.2)	14.0 (12.0–16.0)	14.0 (12.0–17.0)	0.709
Erectile dysfunction (%)	68.1%	77.3%	79.2	74.1%	0.694
IIEF-5	17.0 (8.0–22.0)	11.5 (7.0–20.0)	11.0 (6.7–20.0)	17.0 (9.0–22.0)	0.095
IPSS	6.0 (2.0–11.0)	6.5 (4.0–12.5)	7.0 (3.0–12.5)	5.0 (2.0–10.0)	0.325
AMSS	31.0 (24.0–40.0)	32.0 (29.0–45.0)	34.5 (29.0–40.7)	29.0 (22.0–38.0)	**0.018**
VAI	3.7 (2.4–6.4)	6.5 (3.9–10.8)	6.0 (3.8–8.9)	3.0 (2.2–4.3)	**< 0.001**
TyG	4.9 (4.7–5.1)	5.1 (5.0–5.3)	5.0 (4.8–5.3)	4.8 (4.6–4.9)	**< 0.001**
LAP	50.3 (33.5–83.0)	90.6 (69.9–151.2)	67.2 (49.4–109.6)	39.5 (26.5–54.3)	**< 0.001**
Anti-hypercholesterolemic therapy (%)	59.9%	61.3%	60.2%	54.9%	0.459
Anti-hypertensive therapy (%)	65.8%	64.6%	65.9%	65.5%	0.838
Anti-hyperglycemic drugs (%)	88.9%	87.3%	88.1%	89.6%	0.733

Significant *p* values are in bold

Data are expressed as median (IQR) for continuous variables and as absolute number (%) for categorical variables. Comparison for continuous variables was performed with Mann–Whitney’s test and for categorical variables with Pearson’s Chi-square Test

*AMSS* Aging male symptoms score, *BMI* Body Mass Index, *E2* estradiol, *HbA1c* glycated hemoglobin, *HDL* high density lipoprotein, *IIEF*-*5* International Index of erectile function-5, *IPSS* International Prostatic Symptoms Score, *LAP* lipid accumulation product, *LH* luteinizing hormone, *PSA* prostate specific antigen, *TyG* Triglyceride Glucose Index, *TT* total testosterone, *VAI* Visceral Adiposity Index, *yr* years

VAI, TyG and LAP were all negatively correlated with TT plasma levels (*p* < 0.001) and AMSS score (*p* < 0.02). There was no correlation between these indices and IIEF-5, IPSS, LH, and estradiol.

Seventy-seven patients (77/190, 40.5%) had TT values < 12 nmol/l (Table 1). Of these, 89.8% had low-inappropriately normal LH levels (≤ 9.4 UI/l, hypogonadotropic hypogonadism), and 10.2% had elevated levels (hypergonadotropic hypogonadism).

Compared to patients with normal TT (≥ 12 nmol/l), men with TT < 12 nmol/l had lower 25 OH-vitamin D levels, worse metabolic parameters (BMI, wc, lipid profile, glycaemia, and HbA1c) and higher values of VAI, TyG, LAP and AMSS. As shown in Table 1, patients with frankly low TT (< 8 nmol/l) had higher values of VAI, TyG, and LAP, with respect to men with low TT (8–12 nmol/l), and normal TT (all data *p* < 0.001).

**Fig. 1:**
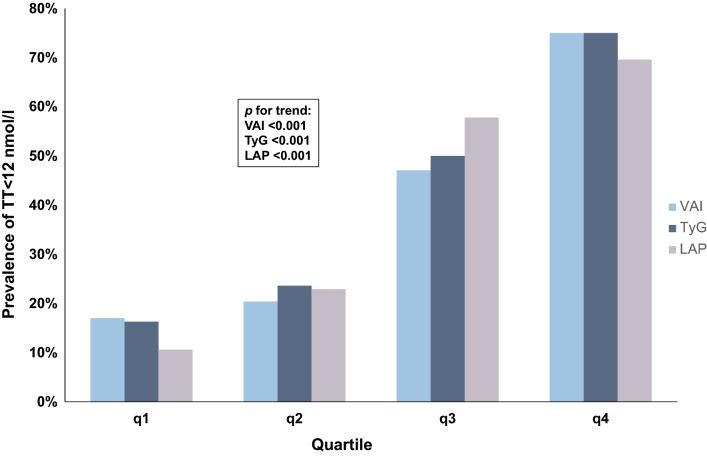
Prevalence of TT < 12 nmol/l according to VAI, TyG and LAP quartiles (their cut-offs are expressed in Table 2). *p* for trend was calculated with Pearson’s Chi-square Test. *LAP* lipid accumulation product, *TyG* Triglyceride Glucose Index, *TT* total testosterone *VAI* Visceral Adiposity Index

Table 2 shows that, as increasing the quartiles of VAI, TyG and LAP, the level of TT decreases (*p* < 0.001), while AMSS score and the prevalence of TT < 8 nmol/l and < 12 nmol/l significantly increase. Figure 1 shows the prevalence of TT < 12 nmol/l in men in the different quartiles of VAI, TyG, and LAP (their cut-offs are expressed in Table 2).

**Table 2:** Comparison of the study population according to VAI, TyG, and LAP quartiles

	VAI < 2.37	2.37 ≤ VAI < 3.68	3.68 ≤ VAI ≤ 6.45	VAI > 6.45	*p* for trend
Age (year)	60.5 (53.0–65.0)	58.0 (51.0–64.7)	58.0 (52.0–64.0)	57.0 (51.0–62.0)	0.502
Creatinine (mg/dl)	0.8 (0.7–0.9)	0.8 (0.7–0.9)	0.9 (0.7–0.9)	0.9 (0.8–1.0)	0.180
25 OH-Vitamin D (nmol/l)	40.5 (24.8–53.5)	37.0 (24.0–51.7)	25.0 (16.0–45.0)	29.0 (18.7–47.0)	**0.028**
LH (IU/l)	4.9 (3.7–5.7)	5.0 (3.8–5.9)	5.7 (3.6–7.7)	5.4 (4.3–7.1)	0.276
E2 (pmol/l)	90.0 (59.0–115.5)	84.0 (56.7–105.0)	75.0 (56.0–97.0)	86.0 (43.0–103.0)	0.622
PSA (ng/ml)	0.8 (0.5–1.8)	1.0 (0.7–1.5)	1.0 (0.5–2.1)	0.8 (0.4–1.1)	0.433
TT (nmol/l)	14.9 (12.2–18.3)	13.8 (12.2–18.1)	12.0 (10.4–15.1)	10.1 (8.3–12.6)	**< 0.001**
TT < 12 nmol/l (%)	17.0%	20.4%	47.1%	75.0%	**< 0.001**
TT < 8 nmol/l (%)	4.2%	9.1%	13.7%	25.0%	**0.024**
Erectile dysfunction (%)	65.0%	58.6%	73.3%	75.0%	0.192
IPSS (index value)	5.0 (2.0–9.0)	4.5 (2.0–9.0)	7.0 (2.0–14.2)	6.0 (3.0–12.0)	0.244
AMSS (index value)	31.0 (23.0–40.0)	26.5 (21.0–36.0)	33.5 (26.2–38.7)	33.0 (29.0–41.0)	**0.018**

	TyG < 4.68	4.68 ≤ TyG < 4.9	4.9 ≤ TyG ≤ 5.11	TyG > 5.11	*p* for trend
Age (years)	58.0 (51.0–63.0)	60.0 (53.0–66.0)	58.0 (52.7–63.2)	55.0 (48.0–63.0)	0.162
Creatinine (mg/dl)	0.8 (0.7–0.9)	0.9 (0.8–1.0)	0.9 (0.7–0.9)	0.9 (0.7–1.0)	**0.034**
25 OH-Vitamin D (nmol/l)	41.0 (23.0–49.0)	34.0 (24.0–50.0)	30.5 (16.7–53.2)	29.0 (16.7–41.0)	0.159
LH (IU/l)	4.9 (3.9–6.0)	4.9 (3.5–6.8)	5.7 (4.0–7.8)	5.4 (4.5–7.1)	0.382
E2 (pmol/l)	91.0 (57.5–123.0)	86.5 (59.7–103.5)	76.0 (50.5–101.7)	82.0 (54.0–100.0)	0.622
PSA (ng/ml)	0.9 (0.5–1.6)	1.0 (0.6–1.6)	0.9 (0.6–1.6)	0.7 (0.4–1.4)	0.522
TT (nmol/l)	15.6 (12.7–18.7)	13.3 (12.0–16.9)	12.0 (10.0–16.5)	9.5 (7.4–11.9)	**< 0.001**
TT < 12 nmol/l (%)	16.3%	23.6%	50.0%	75.0%	**< 0.001**
TT < 8 nmol/l (%)	4.6%	3.6%	16.7%	29.5%	**0.004**
Erectile dysfunction (%)	61.7%	66.7%	65.6%	78.1%	0.226
IPSS (index value)	4.0 (2.0–9.0)	6.0 (2.0–10.0)	7.0 (2.0–12.0)	5.0 (2.0–13.0)	0.671
AMSS (index value)	29.0 (23.0–38.7)	30.5 (22.0–38.0)	31.0 (25.0–39.0)	34.0 (29.0–43.5)	**0.033**

	LAP < 33.54	33.54 ≤ LAP < 50.35	50.35 ≤ LAP ≤ 83.00	LAP > 83.00	*p* for trend
Age (years)	59.5 (53.0–63.0)	57.5 (51.0–65.0)	60.0 (52.2–66.0)	57.0 (49.2–62.0)	0.212
Creatinine (mg/dl)	0.8 (0.7–0.9)	0.8 (0.7–0.9)	0.9 (0.7–1.0)	0.9 (0.8–1.0)	0.064
25 OH-Vitamin D (nmol/l)	37.0 (25.0–50.0)	41.0 (25.0–50.0)	26.5 (16.0–50.7)	27.5 (16.2–41.0)	**0.020**
LH (IU/l)	5.0 (4.0–6.8)	5.0 (3.7–6.8)	5.1 (3.7–6.7)	5.0 (4.1–7.0)	0.981
E2 (pmol/l)	91.0 (68.0–107.5)	81.5 (55.0–112.7)	75.5 (49.0–101.5)	75.0 (45.5–95.0)	0.179
PSA (ng/ml)	1.0 (0.5–1.9)	0.9 (0.5–1.5)	1.0 (0.6–1.6)	0.8 (0.4–1.4)	0.492
TT (nmol/l)	15.1 (12.8–18.6)	14.5 (12.0–19.3)	11.8 (9.9–13.6)	9.9 (7.6–12.9)	**< 0.001**
TT < 12 nmol/l (%)	10.6%	22.9%	57.8%	69.6%	**< 0.001**
TT < 8 nmol/l (%)	2.1%	4.2%	15.5%	30.4%	**0.001**
Erectile dysfunction (%)	58.3%	64.4%	76.3%	73.3%	0.134
IPSS (index value)	5.0 (2.0–9.0)	6.5 (2.0–11.0)	5.0 (2.0–11.0)	6.0 (2.0–13.0)	0.866
AMSS (index value)	28.0 (22.2–36.0)	28.0 (21.7–37.2)	33.5 (27.0–40.7)	34.0 (29.0–41.0)	**0.007**

Significant *p* values are in bold

Data are expressed as median (IQR) for continuous variables and as absolute number (%) for categorical variables. *p* for trend were calculated with Kruskal Wallis test (for continuous variables) and Pearson’s Chi-square Test (for categorical variables)

*AMSS* Aging Male Symptoms Score, *BMI* Body Mass Index, *E2* estradiol, *IPSS* International Prostatic Symptoms Score, *LAP* lipid accumulation product, *LH* luteinizing hormone, *PSA* prostate specific antigen, *TyG* Triglyceride Glucose Index, *TT* total testosterone, *VAI* Visceral Adiposity Index

**Fig. 2:**
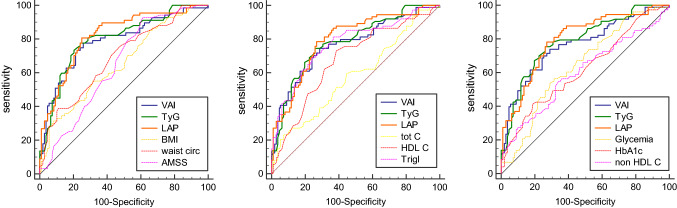
ROC curves for TT < 12 nmol/l: VAI, TyG, and LAP compared with the other parameters. *AMSS* Aging Male Symptoms Score, *BMI* Body Mass Index, *C* cholesterol, *HbA1c* glycated haemoglobin, *HDL* high density lipoprotein, *LAP* lipid accumulation product, *Trigl* triglycerides, *TT* total testosterone, *TyG* Triglyceride Glucose Index, *VAI* Visceral Adiposity Index

We then analyzed the correlation of TT < 12 nmol/l with clinical and biochemical variables (Table 3): a significant positive correlation with AMSS, VAI, TyG and LAP (as well as their constituents BMI, wc, lipid profile, glycemia, HbA1c, total cholesterol, non-HDL-cholesterol, and triglycerides) and a negative correlation with HDL-cholesterol and 25 OH-vitamin D levels were found. A binary linear logistic regression analysis was performed to assess the factors related with TT < 12 nmol/l, both considering the 3 indices (VAI, TyG and LAP) (Table 4) and their constituent parameters (Supplementary table S1). At these analyses, the main factors associated with TT < 12 nmol/l were VAI, TyG, LAP, wc, triglycerides, and AMSS. Interestingly, the correlation of the three indices with TT < 12 nmol/l was in general higher than their respective constituents. Therefore, ROC curves were performed to determine the significant predictive parameters of TT < 12 nmol/l (Fig. 2, Supplementary table S2). There was no significant difference between the three parameters (VAI, TyG and LAP) as a predictive index of TT < 12 nmol/l. The three indices were instead significantly more predictive of TT < 12 nmol/l than the other parameters (BMI, wc, lipid profile, glycemia, HbA1c, AMSS), except for triglycerides (and wc for VAI). Triglycerides ≥ 113.0 mg/dl predicted TT < 12 nmol/l with good sensitivity (80.5%) but moderate specificity (62.8%). On the contrary, VAI ≥ 3.985, TyG ≥ 4.925 and LAP ≥ 51.645 had good sensitivity (74.3%, 74.7% and 78.4%, respectively) and specificity (71.4%, 73.5% and 73.2%, respectively) in the diagnosis of male hypogonadism.

**Table 3:** Correlations of TT < 12 nmol/l with the different variables. Significant *p* values are in bold

	TT < 12 nmol/l	
	*r*	*p* value
Age	− 0.044	0.549
BMI	0.271	**< 0.001**
Waist circumference	0.317	**< 0.001**
Glycemia	0.197	**0.006**
HbA1c	0.234	**0.001**
Total cholesterol	0.173	**0.017**
HDL cholesterol	− 0.278	**< 0.001**
Non-HDL cholesterol	0.212	**0.003**
Triglycerides	0.418	**< 0.001**
Creatinine	0.205	**0.012**
LH	0.138	0.063
E2	− 0.085	0.261
PSA	0.122	0.345
25 OH-Vitamin D	− 0.153	**0.038**
Right testicular volume	0.023	0.759
Left testicular volume	− 0.027	0.714
Erectile dysfunction	0.051	0.496
IIEF-5	− 0.143	0.053
IPSS	0.079	0.296
AMSS	0.226	**0.003**
VAI	0.361	**< 0.001**
TyG	0.464	**< 0.001**
LAP	0.426	**< 0.001**
Anti-hypercholesterolemic therapy	0.056	0.443
Anti-hypertensive therapy	0.022	0.770
Anti-hyperglycemic drugs	0.039	0.723

Pearson’s correlations (for continuous variables) and Point-Biserial correlations (for dichotomous variables) were performed

*AMSS* Aging male symptoms score, *BMI* Body Mass Index, *E2* estradiol, *HbA1c* glycated hemoglobin, *HDL* high density lipoprotein, *IIEF*-*5* International Index of erectile function-5, *IPSS* International Prostatic Symptoms Score, *LAP* lipid accumulation product, *LH* luteinizing hormone, *PSA* prostate specific antigen, *TyG* Triglyceride Glucose Index, *TT* total testosterone, *VAI* Visceral Adiposity Index

**Table 4:** Binary linear logistic regression on the factors associated with TT < 12 nmol/l, considering the three indices VAI, TyG, and LAP. Significant *p* values are in bold

	TT < 12 nmol/l	
	OR (CI)	*p*
VAI	1.439 (1.215–1.703)	**< 0.001**
Glycemia	1.004 (0.992–1.016)	0.507
HbA1c	0.905 (0.565–1.449)	0.678
Total cholesterol	0.996 (0.977–1.016)	0.690
Non-HDL cholesterol	1.008 (0.989–1.027)	0.411
Creatinine	4.161 (0.505–34.315)	0.185
25 OH-Vitamin D	0.972 (0.949–0.996)	**0.023**
AMSS	1.056 (1.009–1.106)	**0.020**
TyG	164.670 (11.996–2260.529)	**< 0.001**
Age	1.041 (0.985–1.100)	0.152
BMI	0.858 (0.682–1.079)	0.190
Waist circ	1.125 (1.018–1.243)	**0.021**
HbA1c	0.732 (0.476–1.125)	0.155
Total cholesterol	0.986 (0.960–1.012)	0.275
HDL cholesterol	0.984 (0.939–1.031)	0.500
Non-HDL cholesterol	1.016 (0.993–1.039)	0.182
Creatinine	2.049 (0.191–22.033)	0.554
25 OH Vit. D	0.979 (0.955–1.004)	0.095
AMSS	1.057 (1.005–1.113)	**0.032**
LAP	1.039 (1.017–1.062)	**< 0.001**
Age	1.052 (0.997–1.110)	0.063
BMI	0.965 (0.866–1.076)	0.523
Glycemia	1.004 (0.992–1.016)	0.545
HbA1c	0.971 (0.603–1.562)	0.902
Total cholesterol	0.982 (0.951–1.013)	0.256
HDL cholesterol	0.989 (0.942–1.038)	0.653
Non-HDL cholesterol	1.021 (0.992–1.051)	0.158
Creatinine	2.447 (0.242–24.767)	0.449
25 OH Vit. D	0.975 (0.950–1.001)	0.057
AMSS	1.053 (1.003–1.106)	**0.037**

*AMSS* Aging male symptoms score, *BMI* Body Mass Index, *CI* confidence intervals, *HbA1c* glycated hemoglobin, *HDL* high density lipoprotein, *LAP* lipid accumulation product, *OR* odds ratio, *TyG* Triglyceride Glucose Index, *TT* total testosterone, *VAI* Visceral Adiposity Index

## Discussion

This is the first study evaluating the association of the common cardiometabolic indices VAI, TyG and LAP with hypogonadism in men with type 2 diabetes. We found that these parameters were all strongly negatively associated with TT levels, and the prevalence of hypogonadism increased as quartiles of VAI, TyG and LAP increased, reaching up to ~ 70.0–75.0% in men with the highest values. All three indices were also correlated with AMSS score, confirming their association with biochemical (T levels) and clinical (symptoms) hypogonadism. Furthermore, VAI, TyG, and LAP could predict hypogonadism with good reliability, higher than their respective components and classic clinical parameters, as BMI, waist circumference and AMSS score. Finally, we identified cut-off values for these indices associated with low T with good sensitivity and specificity.

The bilinear relationship between type 2 diabetes and male hypogonadism has been studied extensively and well explained [6, 28]. Up to 40% of men with type 2 diabetes may have T deficiency, which is generally associated with low-inappropriately normal gonadotropins (secondary or normogonadotropic hypogonadism), due to functional alterations of the gonadal axis from metabolic disease [28]. Our data agree with these findings, as we found a prevalence of biochemical hypogonadism (TT < 12 nmol/l) of 40.5% and clinical hypogonadism of 68.1% (ED prevalence at IIEF-5) and 69.3% (by AMSS score). Low T was associated in 90% of cases with average/low LH levels, and patients with frankly low TT (< 8 nmol/l) were 13.2%.

Noteworthy, in men with type 2 diabetes, hypogonadism has been associated with worse disease control and outcomes, such as cardiovascular complications and prognosis, and studies showed that it predicts metabolic syndrome and type 2 diabetes [6, 10, 29]. Testosterone deficiency—in diabetic and non-diabetic patients—has indeed been associated with worse anthropometric parameters and glyco-metabolic profile [28], lower levels of vitamin D [30] and higher AMSS [26], in agreement with the findings of the present study. Significantly, testosterone replacement therapy in hypogonadal diabetic patients could improve the glyco-metabolic profile, disease control, and prognosis [8, 10, 31]. Therefore, identifying hypogonadism in patients with type 2 diabetes is of primary importance to better manage these patients and improve prognosis and quality of life [32, 33]. However, in routine clinical practice, this aspect is often neglected, although the possible benefits for the patients have been well described [12]. It has been suggested that sexual symptoms and testosterone levels should sound as the harbinger for further andrologic and cardiovascular investigation and that diabetologists have the chance to have a symptom (ED) that help them in better management of patients, their comorbidities and complications [12]. However, ED in the setting of diabetic men might have different etiologies than low T and might represent an unspecific symptom overlapping with other comorbidities [13]. Indeed, in our study, the prevalence of ED did not differ significantly between hypogonadal and non-hypogonadal patients.

Our study identified VAI, TyG and LAP as sensitive predictors of hypogonadism in men with type 2 diabetes, therefore suggesting their routine use to direct the patient to andrological evaluation. Previous studies in males with type 2 diabetes or metabolic syndrome showed that higher values of one or more of these three indices express worst anthropometric parameters, poorer glyco-metabolic control, and elevated cardiometabolic risk and more diabetic vascular complications [18, 34–37]. In addition, TyG and LAP have been observed as predictors of hypogonadism in middle-elderly male general Chinese population [22, 23], and VAI as a marker of cardiometabolic risk in men with congenital hypogonadotropic hypogonadism [38]. Recently, LAP has also been shown as a predictor of worst seminal quality in infertile men [39].

In general, higher values of these cardiometabolic indices are sign of worst anthropometric and glyco-metabolic control and, therefore of increased risk of functional impairment of the gonadal axis [5]. Increased adiposity and metabolic dysfunction cause alteration of the gonadal axis through mechanisms mainly mediated by insulin resistance, adipokines, leptin and estrogens, which are elevated in men with overweight/obesity due to increased aromatization of T in the adipose tissue [40, 41]. Increased insulin levels, leptin, estrogens and pro-inflammatory adipokines lead to functional suppression of the hypothalamic-pituitary–testicular axis mainly through inhibition of gonadotropin production [41]. In addition, insulin resistance reduces the levels of SHBG, resulting in a decrease in TT levels [6]. Although our study did not consider the measurement of insulin resistance, leptin, and SHBG (to calculate free T), wc, BMI, and estradiol levels were elevated, supporting this pathophysiologic pathway.

Apart from these limitations, the strength of our study is the identification for the first time in men with type 2 diabetes of the diagnostic utility of VAI, TyG and LAP, which are simple to evaluate, in suspecting hypogonadism, and the identification of cut-offs values for these indices strongly predictive of reduced T levels. In particular, we suggest an andrological evaluation, to assess male hypogonadism, in diabetic patients with values of VAI >  ~ 3.98 or TyG >  ~ 4.9 or LAP >  ~ 51.6. For this purpose, the latter could be of quick and better clinical use than traditional metabolic parameters and sexual symptoms.

In conclusion, the use of these cardiometabolic parameters could facilitate the collaboration between diabetologists and andrologists, to improve a shared management of men with hypogonadism and type 2 diabetes.

## Supplementary Information

Below is the link to the electronic supplementary material.

Supplementary file1 (DOCX 25 KB)
