# Supplementary material for: Cardiometabolic indices predict hypogonadism in male patients with type 2 diabetes
Source: J Endocrinol Invest. 2022 Oct 25;46(3):599–608. doi: 10.1007/s40618-022-01941-0 (PMC9938038; doi:10.1007/s40618-022-01941-0)
Supplement: Supplementary file 1 — Supplementary file1 (DOCX 25 KB) [file 40618_2022_1941_MOESM1_ESM.docx]

**Supplementary table S1.** Binary linear logistic regression on the factors associated with TT <12 nmol/l, considering the constituents (in italic) of VAI (**a**), TyG (**b**), and LAP (**c**). Significant *p* values are in bold.

|  | **TT <12 nmol/l** | |
| --- | --- | --- |
|  | **OR (CI)** | ***p*** |
| **a**  *Age*  *BMI*  *Waist circumference*  *HDL cholesterol*  *Triglycerides*  Glycemia  HbA1c  Total cholesterol  Non-HDL cholesterol  Creatinine  25 OH-Vitamin D  AMSS | 1.054 (0.996-0.116)  0.843 (0.668-1.062)  1.126 (1.017-1.246)  0.993 (0.942-1.046)  1.021 (1.010-1.032)  1.005 (0.992-1.018)  0.916 (0.550-1.526)  0.978 (0.946-1.011)  1.022 (0.991-1.054)  2.229 (0.197-26.834)  0.975 (0.949-1.001)  1.053 (1.001-1.107) | 0.067  0.147  **0.022**  0.778  **<0.001**  0.424  0.737  0.196  0.162  0.507  0.064  **0.045** |
| **b** |  |  |
| *Triglycerides*  *Glycemia*  Age  BMI  Waist circumference  HbA1c  Total cholesterol  HDL cholesterol  Non-HDL cholesterol  Creatinine  25 OH Vit. D  AMSS | 1.021 (1.010-1.032)  1.005 (0.992-1.018)  1.054 (0.996-0.116)  0.843 (0.668-1.062)  1.126 (1.017-1.246)  0.916 (0.550-1.526)  0.978 (0.946-1.011)  0.993 (0.942-1.046)  1.022 (0.991-1.054)  2.229 (0.197-26.834)  0.975 (0.949-1.001)  1.053 (1.001-1.107) | **<0.001**  0.424  0.067  0.147  **0.022**  0.737  0.196  0.778  0.162  0.507  0.064  **0.045** |
| **c** |  |  |
| *Triglycerides*  *Waist circumference*  Age  BMI  Glycemia  HbA1c  Total cholesterol  HDL cholesterol  Non-HDL cholesterol  Creatinine  25 OH Vit. D  AMSS | 1.021 (1.010-1.032)  1.126 (1.017-1.246)  1.054 (0.996-0.116)  0.843 (0.668-1.062)  1.005 (0.992-1.018)  0.916 (0.550-1.526)  0.978 (0.946-1.011)  0.993 (0.942-1.046)  1.022 (0.991-1.054)  2.229 (0.197-26.834)  0.975 (0.949-1.001)  1.053 (1.001-1.107) | **<0.001**  **0.022**  0.067  0.147  0.424  0.737  0.196  0.778  0.162  0.507  0.064  **0.045** |

AMSS= Aging male symptoms score; BMI= Body Mass Index; CI= confidence intervals; HbA1c= glycated haemoglobin; HDL= High Density Lipoprotein; LAP =Lypid Accumulation Product; OR= Odds ratio; TyG= Triglyceride glucose index; TT= Total Testosterone; VAI= Visceral Adiposity Index.

**Supplementary table S2.** Analysis of ROC curves for TT <12 nmol/l: comparison of the diagnostic accuracy between VAI, TyG, LAP and the other parameters. Significant *p* values are in bold.

|  | **AUC (CI)** | ***p* value^1^** | **Difference, *p* value^2^** |
| --- | --- | --- | --- |
| VAI | 0.767 (0.695-0.838) | **<0.001** | VAI-TyG: z=0.598, p=0.549  VAI-LAP: z=1.245, p=0.213  TyG-LAP: z=0.593, p=0.553  VAI-BMI: z=2.584, **p=0.009**; VAI-waist circumference: z=1.943, p=0.052; VAI-AMSS: z=2.496, **p= 0.012**; VAI-glycemia: z=2.262, **p=0.023**; VAI-HbA1c: z=3.010, **p=0.003**; VAI-non-HDL cholesterol: z=3.038, **p=0.024**; VAI-Total cholesterol: z=3.661, **p<0.001**; VAI-HDL cholesterol: z=2.699, **p=0.007**; VAI-Triglycerides: z=0.397, p=0.691  TyG-BMI: z=2.794, **p=0.005**; TyG-waist circumference: z=2.175, **p=0.029**; TyG-AMSS: z=2.854, **p=0.004**; TyG-glycemia: z=3.683, **p<0.001**; TyG-HbA1c: z=3.941, **p<0.001**; TyG-non-HDL cholesterol: z=3.677, **p<0.001**; TyG-Total cholesterol: z=4.490, **p<0.001**; TyG-HDL cholesterol: z=2.276, **p=0.023**; TyG-Triglycerides: z=0.412, p=0.680  LAP-BMI: z=4.455, **p<0.001**; LAP-waist circumference: z=3.725, **p=0.002**; LAP-AMSS: z= 3.337, **p<0.001**; LAP-glycemia: z=2.916, **p=0.003**; LAP-HbA1c: z=4.012, **p<0.001**; LAP-non-HDL cholesterol: z=3.833, **p<0.001**; LAP-Total cholesterol: z=4.770, **p<0.001**; LAP-HDL cholesterol: z=2.681, **p=0.007**; LAP-Triglycerides: z=1.227, p=0.219 |
| TyG | 0.781 (0.715-0.839) | **<0.001** |  |
| LAP | 0.798 (0.732-0.863) | **<0.001** |  |
| BMI | 0.648 (0.579-0.725) | **<0.001** |  |
| Waist circumference | 0.679 (0.615-0.757) | **<0.001** |  |
| AMSS | 0.648 (0.572-0.718) | **<0.001** |  |
| Glycemia | 0.623 (0.569-0.702) | **0.004** |  |
| HbA1c | 0.620 (0.545-0.690) | **0.003** |  |
| Non-HDL cholesterol | 0.591 (0.526-0.662) | **0.037** |  |
| Total cholesterol | 0.564 (0.506-0.642) | 0.141 |  |
| HDL cholesterol | 0.677 (0.605-0.744) | **<0.001** |  |
| Triglycerides | 0.773 (0.706-0.831) | **<0.001** |  |

^1^the diagnostic value for ROC

^2^comparison of AUC between the parameters (z test)

AMSS= Aging male symptoms score; AUC= are aunder the curve; BMI= Body Mass Index; CI= confidence interval; HbA1c= glycated haemoglobin; HDL= High Density Lipoprotein; LAP =Lypid Accumulation Product; TyG= Triglyceride glucose index; VAI= Visceral Adiposity Index
